# Roles of PLODs in Collagen Synthesis and Cancer Progression

**DOI:** 10.3389/fcell.2018.00066

**Published:** 2018-06-28

**Authors:** Yifei Qi, Ren Xu

**Affiliations:** ^1^Markey Cancer Center, University of Kentucky, Lexington, KY, United States; ^2^Department of Pharmacology and Nutritional Sciences, University of Kentucky, Lexington, KY, United States

**Keywords:** collagen, extracellular matrix, lysyl hydroxylation, procollagen-lysine 2-oxoglutarate 5-dioxygenase, cancer progression

## Abstract

Collagen is the major component of extracellular matrix. Collagen cross-link and deposition depend on lysyl hydroxylation, which is catalyzed by procollagen-lysine, 2-oxoglutarate 5-dioxygenase (PLOD). Aberrant lysyl hydroxylation and collagen cross-link contributes to the progression of many collagen-related diseases, such as fibrosis and cancer. Three lysyl hydroxylases (LH1, LH2, and LH3) are identified, encoded by *PLOD1, PLOD2*, and *PLOD3* genes. Expression of PLODs is regulated by multiple cytokines, transcription factors and microRNAs. Dysregulation of PLODs promotes cancer progression and metastasis, suggesting that targeting PLODs is potential strategy for cancer treatment. Here, we summarize the recent progress in the investigation of function and regulation of PLODs in normal tissue development and disease progression, especially in cancer.

## Introduction

Collagen is one of the major components of extracellular matrix. The collagen-cell interaction induces biochemical and biophysical signals, which is essentially for normal tissue function and cancer progression (Egeblad et al., 2010; Xiong and Xu, 2016). The collagen family contains 28 members (Heino, 2007) and can be divided into two groups: fibrillar collagen (type I, II, III, V, XI) and non-fibrillar collagen (type IV, VIII, X, IX, XII, XIV, XV, XVIII, XIX, XXI). Collagen is the most abundant protein in our body, and presents in both normal tissues and cancer. Type I collagen, the most common type fibrillar, has been identified in many tissues, including skin, tendon, vascular ligature and bone; while type II collagen is the main collagenous component of cartilage. Non-fibrillar type IV collagen is required for basement membrane formation (Paulsson, 1992). Cell-collagen interaction induces cellular signaling via integrin [included α1β1 (Tulla et al., 2001; Hamaia et al., 2012), α2β1 (Tulla et al., 2001; Carafoli et al., 2013), α10β1 (Camper et al., 1998) and α11β1 (Tiger et al., 2001; Hamaia et al., 2012)], discoidin domain receptors (Leitinger, 2003, 2011) and Leukocyte-Associated Immunoglobulin-Like Receptor-1 (Rygiel et al., 2011; Kim et al., 2017). Collagen regulates tumor progression by modulating cancer cell migration, invasion (Xiong et al., 2014), proliferation (Pollard, 2004), survival (Cheon et al., 2014) and metastasis (Oudin et al., 2016; Sun et al., 2016).

All collagen is composed of a triple helix, and the most common motif of the triple helix sequence is Gly-X-Y (X and Y represent proline or hydroxyproline) (Albaugh et al., 2017). Collagen is synthesized in the rough endoplasmic reticulum (ER) as a precursor (Nimni, 1983). After peptide bond formation, proline and lysyl hydroxylation is catalyzed by prolyl 4-hydroxylase (P4H) and procollagen-lysine,2-oxoglutarate 5-dioxygenase (PLOD). The hydroxylation of lysyl residues is one of the critical steps of collagens biosynthesis. It usually occurs in the Y position of the repeating Gly-X-Y motif (Barnes et al., 1974; Valtavaara et al., 1998). Three PLODs (PLOD1, PLOD2 and PLOD3) has been identified, catalyzing the lysyl hydroxylation to hydroxylysine (Hausmann, 1967; Rhoads and Udenfriend, 1968; Kivirikko Ki, 1998; Rautavuoma et al., 2004).

Hydroxylysine residue is critical for the formation of covalent cross-links and collagen glycosylation (Valtavaara et al., 1998). PLODs catalyze hydroxylation of lysine (Lys) intracellularly before collagen is secreted, and then lysyl oxidase (LOX) binds to hydroxylysine (Hyl) residues in the extracellular collagen fibers and induces the cross-link formation (Saito and Marumo, 2010). This enzyme dependent collagen crosslinking stabilizes newly formed collagen fibers and enhances the stiffness of the matrix. During collagen maturation, the hydroxylysine residues in the helix region are often modified by the O-linked glycosylation. These reactions are catalyzed by hydroxylysine galactosyltransferase (GT) and galactosylhydroxylysine -glucosyltransferase (GGT) (Shinkai and Yonemasu, 1979; Yamauchi and Sricholpech, 2012). The enzymatic activities of GT and GGT are found in multifunctional PLOD3, but not in PLOD1 and PLOD2 (Heikkinen et al., 2000). Mutations of the human *PLOD3* gene lead to congenital disorders affecting connective tissues of various organs (Salo et al., 2008), suggesting that GGT activity of PLOD3 is crucial for the normal function of collagen.

The mutation or overexpression of PLODs has been detected in many human diseases. The kyphoscoliotic type of Ehlers-Danlos syndrome (EDS type VIA) is due to a mutation in the *PLOD1* gene (Rohrbach et al., 2011; Zahed-Cheikh et al., 2017). The reduction of PLOD3 protein at the basement membrane is associated with recessive dystrophic epidermolysis bullosa (RDEB) progression (Watt et al., 2015). The overexpression of PLOD2 is detected in many types of cancer. Therefore, investigating the function and the regulation of PLODs in normal organ development and disease progression may identify potential targets for the treatment of collagen-related diseases.

### Structure of PLODs

**Figure 1:**
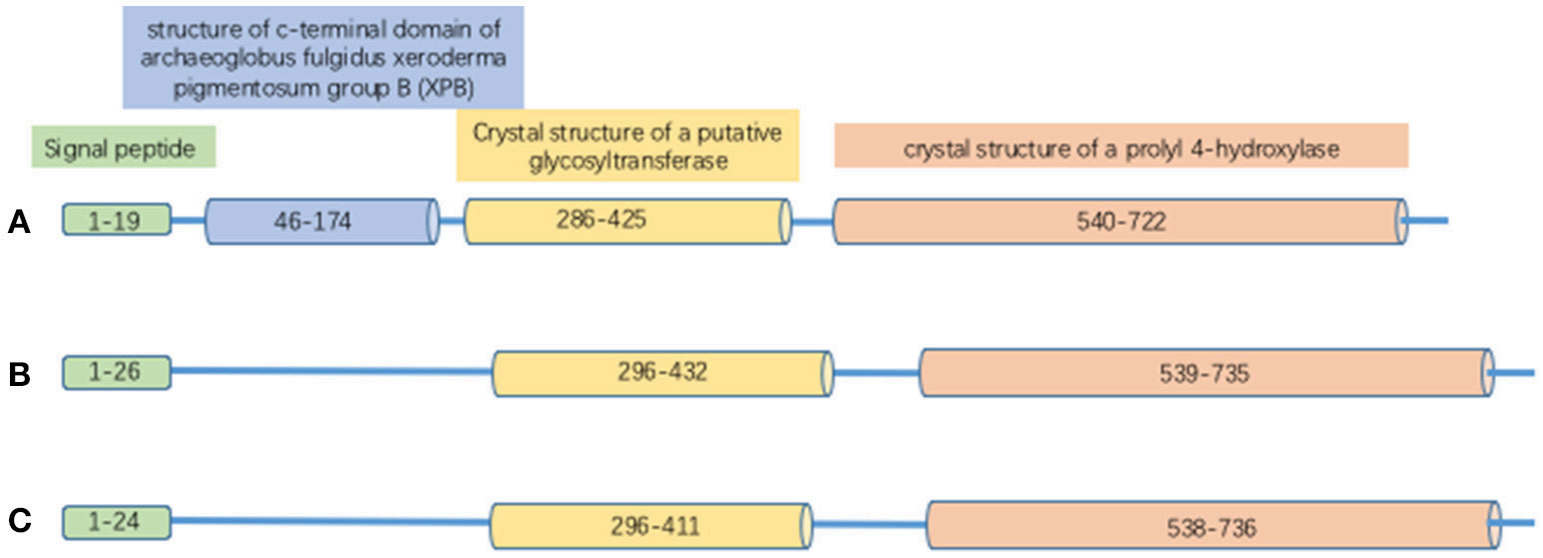
The structure of PLOD proteins. **(A)** PLOD1; **(B)** PLOD2; **(C)** PLOD3.

Proteins in the PLOD family are highly homologous; the overall identity in protein sequences among PLOD1, 2 and 3 is 47% (Valtavaara et al., 1998). PLOD protein has binding sites for cofactor Fe^2+^ and L-ascorbate. It also contains 26 amino acid signal peptide and a Prolyl 4-hydroxylase alpha subunit homologs domain (Figure 1). *PLOD1* gene locates on chromosome 1p36 (Tasker et al., 2006) and is composed by 19 exons (Giunta et al., 2005). Collagen hydroxylation catalyzed by PLOD1 is crucial for bone mineral density (BMD) and bone quality (Tasker et al., 2006). *PLOD2* gene is at chromosome 3q23-q24 (Szpirer et al., 1997) and also contains 19 exons. Two splice variants (LH2a and LH2b) have been identified in the *PLOD2* gene; LH2b differs from LH2a by incorporating the small exon 13A (Valtavaara, 1999). PLOD2 plays a key role in formation of stabilized collagen cross-links (Gilkes et al., 2013a). *PLOD3* gene is localized to chromosome 7q36 (Hautala et al., 1992; Szpirer et al., 1997; Valtavaara et al., 1998), and PLOD3 activity is important for the biosynthesis of type IV and VI collagen (Rautavuoma et al., 2004; Sipilä et al., 2007). PLOD1 and PLOD3 hydroxylate lysyl residues in the collagen triple helix, whereas PLOD2 (LH2b) hydroxylate lysyl residues in the telopeptides of collagen (Valtavaara, 1999). PLOD3 has glycosylation activity that induces either monosaccharide or disaccharide attaching to collagen hydroxylysines (Valtavaara, 1999).

### Regulation of PLOD expression

**Figure 2:**
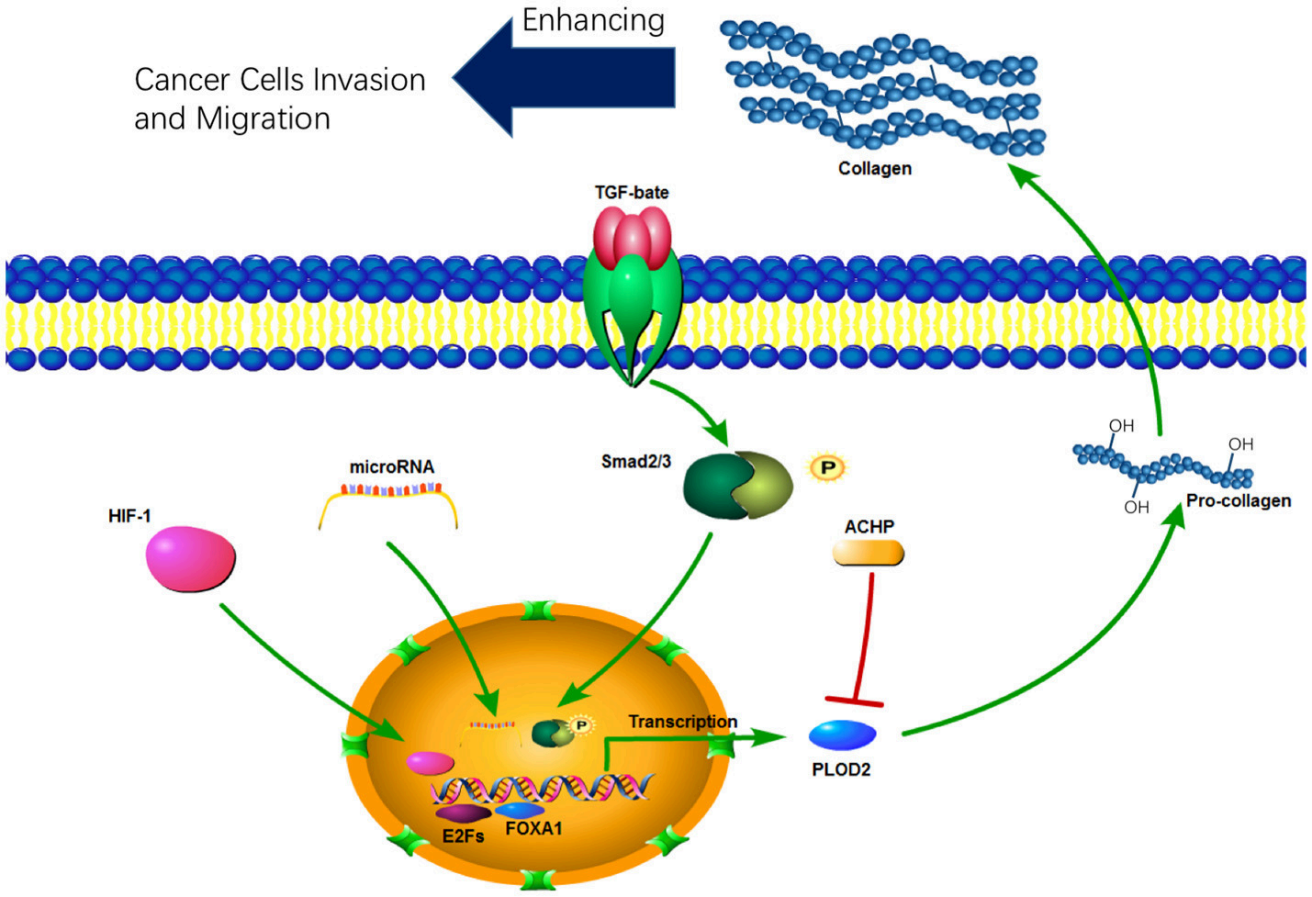
The function and regulation of PLOD2 in cancer progression.

PLOD expression is mainly regulated at the transcription level. A number of cytokines, signaling pathways, and microRNAs have been identified to be involved in transcriptional regulation of PLODs (Table 1). PLOD2 is induced by hypoxia-inducible factor-1α (HIF-1α) under hypoxia condition, which in turn enhance hypoxia-induced Epithelial-Mesenchymal Transition (EMT) phenotypes in glioma cells (Song et al., 2017) and breast cancer cells (Gilkes et al., 2013b). In addition, hypoxia-inducible factor 1 (HIF-1) also activates transcription of PLOD1 in breast cancer cells; however, function PLOD2 is more important for HIF-1-induced cancer progression (Gilkes et al., 2013b). PLOD2 is also directly regulated by miR-26a-5p and miR-26b-5p, and PLOD2 expression is a potential prognostic marker for patients with bladder cancer (Miyamoto et al., 2016) and renal cell carcinoma (Kurozumi et al., 2016). TGF-β signaling is another important regulator of PLOD2 expression (Remst et al., 2014). SP1 and SMAD3, as downstream targets of TGF-β signaling, recruit histone modifying enzymes to the *PLOD2* promoter region and induced PLOD2 transcription (Gjaltema et al., 2015). In addition, transcription factor E2Fs (Hollern et al., 2014) and FOXA1 (Du et al., 2017) have been identified as regulators of PLOD2 during cancer progression (Figure 2).

**Table 1:** The regulation of PLODs.

**PLODs**	**Regulated by**	**Tissues or cell lines**	**Results**
PLOD1	ACHP	Dermal fibroblasts	Suppression
	BMP-2	AT-MSCs	Early upregulated, later downregulated
	TGF-β	AT-MSCs	Early upregulated, later downregulated
	HIF-1α	Hypoxic breast cancer cells	Upregulated
	PITX2	A variety of tissues	Upregulated
PLOD2	ACHP	Dermal fibroblasts	Suppression
	HIF-1α	Glioma cell, hypoxic breast cancer cells	Upregulated
	miR-26a-5p and miR-26b-5p	Bladder cancer, renal cell carcinoma	Upregulated
	TGF-β	Human synovial fibroblasts	Upregulated
	E2Fs	NSCLC, Mouse Model of Metastatic Breast Cancer	Upregulated
	FOXA1	NSCLC	Upregulated
	ER complex of resident chaperones	Dermal fibroblast	Upregulated the activity
PLOD3	ACHP	Dermal fibroblasts	Suppression
	BMP-2	AT-MSCs	Downregulated
	TGF-beta1	AT-MSCs	Downregulated
	miR-663a	Human hepatoma Huh7 cells, Hek 293 cells and Hela cells	Downregulated

Regulation of PLOD1 and PLOD3 expression is not well-investigated compared to PLOD2. Differential display analysis identified *PLOD1* as a potential target gene of TNFα in human chondrocyte-like cells (Ah-Kim et al., 2000). Using chromatin immunoprecipitation and luciferase reporter assay, Hjalt showed that PITX2 directly regulates PLOD1 expression by binding to the promoter region. Inactivation of this pathway may cause the Rieger syndrome (Hjalt et al., 2001). One report show that miR-663a reduces PLOD3 expression by targeting to 3'-UTR of PLOD3 mRNA, subsequently reducing extracellular accumulation of type IV collagen (Amodio et al., 2016).

ACHP (2-Amino-6-[2-(cyclopropylmethoxy)-6-hydroxyphenyl]-4-(4-piperidinyl)−3 pyridinecarbonitrile), a selective inhibitor of IκB kinase, suppresses expression of all three *PLOD* genes in dermal fibroblasts, but not in lung fibroblasts (Mia and Bank, 2015). Therefore, activation of NF-kB pathway may induce PLOD expression in certain types of cells. Treatment with bone morphogenetic protein-2 (BMP-2) and TGF-β1 induces PLOD1 expression in adipose tissue-derived mesenchymal stem cells (AT-MSCs). Interestingly, neither BMP-2 nor TGF-β1 can induce PLOD2 expression (Knippenberg et al., 2009). Given the crucial function of PLODs in collagen synthesis, further defining the molecular mechanisms by which PLOD expression is regulated may significantly expand our understanding of collagen-related disease progression.

## Physiological functions of PLODs

Collagen is the major component of connective tissues and maintains the structural integrity and the stability of tissues and organs (Patino et al., 2002). The hydroxylysine residues provide attachment sites for the carbohydrates and tensile strength and mechanical stability for the collagen fibrils (Rautavuoma et al., 2004). The abnormal expression or mutation of PLODs is associated with collagen-related diseases, such as Kyphoscoliotic type of EDS VIA (Pousi et al., 1994; Giunta et al., 2005; Abdalla et al., 2015; van Dijk et al., 2017; Zahed-Cheikh et al., 2017), Bruck Syndrome (BS) (Gistelinck et al., 2016) and RDEB (Watt et al., 2015) (Table 2). PLOD1 regulates the hydroxylation of lysyl residues on collagen type V. The duplication of the exon 10 to exon 16 region of *PLOD1* (p.Glu326_Lys585dup) gene (Pousi et al., 1994; Giunta et al., 2005) and two mutations on Gln208 and Tyr675 cause the loss function of PLOD1, which may lead to EDS VIA (Abdalla et al., 2015; van Dijk et al., 2017). In addition, *PLOD1* has been identified as a susceptibility gene for reduced BMD (Tasker et al., 2006; Yamada and Shimokata, 2007).

**Table 2:** The association of PLODs with human diseases.

**PLOD protein family**	**Collagen substrate**	**Human disease**
PLOD1	Type V	EDS VIA (Pousi et al., 1994; Giunta et al., 2005; Abdalla et al., 2015; van Dijk et al., 2017; Zahed-Cheikh et al., 2017) BMD (Tasker et al., 2006; Yamada and Shimokata, 2007) Early Alzheimer's disease (Chong et al., 2013)
PLOD2	Type I (Gistelinck et al., 2016)	bAVM (Neyazi et al., 2017) BS (Gistelinck et al., 2016) Carcinoma (Conklin et al., 2011; Rajkumar et al., 2011; Noda et al., 2012; Gilkes et al., 2013b; Li et al., 2017; Song et al., 2017)
PLOD3	Type IV and VI (Sipilä et al., 2007) type I (Sricholpech et al., 2011)	Recessive dystrophic epidermolysis bullosa (RDEB) (Watt et al., 2015)

Dysregulation of PLOD2 is associated with brain arteriovenous malformations and cancer progression. PLOD2 is overexpressed in brain arteriovenous malformations (bAVM), and the levels of PLOD2 expression correlated with bAVM size (Neyazi et al., 2017). PLOD2 mutant zebrafish display molecular and tissue abnormalities in the musculoskeletal system that are concordant with clinical findings in BS patients (Gistelinck et al., 2016). There is evidence that the levels of mature hydroxylysine aldehyde-derived cross-links typical for skeletal tissues is increased in vein graft disease, this is accompanied by upregulation of PLOD2 (Kahle et al., 2016). Furthermore, increased PLOD2 expression has been detected in the macroscopically injured region of the capsule, and upregulation of TGF-β1, TGFβR1, and PLOD2 is likely related to the disease progression (Belangero et al., 2016).

It has been shown that PLOD3 mutations are associated with the connective tissue disorder (Salo et al., 2008). Analysis of PLOD3 knock-out embryos and cells indicate that loss of PLOD2 reduces glycosylated hydroxylysines on type IV and VI collagen with abnormal distribution (Sipilä et al., 2007). Reduced glycosylation may inhibit the tetramerization and secretion of type VI collagen. Another function of PLOD3 is to glucosylate galactosylhydroxylysine residues in type I collagen in osteoblasts. The G-Hyl glucosylation induced by PLOD3 is crucial for collagen fibrillogenesis *in vitro* (Sricholpech et al., 2011).

### PLODs in cancer progression and metastasis

Increased collagen deposition and cross-linking promote cancer development and progression by enhancing cancer cell migration, invasion and proliferation (Provenzano et al., 2006, 2008; Levental et al., 2009; Zhu et al., 2015). Therefore, PLODs may contribute to cancer progression by modulating collagen cross-link and maturation.

Increased PLOD expression has been detected in many types of cancer. The PLOD2 expression level is significantly upregulated in breast cancer compared to normal mammary tissue, and the upregulation correlates with short disease-related survival (Gjaltema et al., 2015). In esophageal squamous-cell carcinoma (ESCC), expression of the tumor suppressor gene esophageal cancer-related gene 4 has a negative association with PLOD1 and PLOD2 (Li et al., 2017). The PLOD2 expression is significantly correlated disease-free survival and tumor size in hepatocellular carcinoma (HCC) (Noda et al., 2012). PLOD3 is overexpressed in HCC (Elsemman et al., 2016; Shen et al., 2018) and is a potential diagnosis marker for early-stage HCC (Shen et al., 2018). Knockdown of PLOD3 suppresses liver tumor incidence as well as tumor growth rates in spontaneous mouse HCC model (Shen et al., 2018). Nicastri used a quantitative proteomic technique and identified 54 up-regulated glycoproteins in colorectal cancer samples, including PLOD2 and PLOD3 (Nicastri et al., 2014).

Increased PLOD2 expression is crucial for tumor invasion and metastasis (Figure 2). For instance, silencing PLOD2 expression in the breast cancer cell line MDA-MB 231 reduces cancer metastasis and collagen deposition in the primary tumor tissue; interestingly, PLOD2 expression has little effect on the primary tumor growth (Gilkes et al., 2013b). Hypoxia- and TGF-β1-induced PLOD2 expression promotes the migratory, invasive and adhesive capacities of cervical cancer cells by promoting EMT and the formation of focal adhesion (Remst et al., 2014; Xu et al., 2017). In HIF-1α-deficient tumors, ectopic PLOD2 expression restores the migration and metastatic potential, and inhibition of PLOD2 activity suppresses the tumor metastases (Eisinger-Mathason et al., 2013). Although HIF-1 induces expression of PLOD1 and PLOD2, PLOD2 expression in breast cancer cells is more important for fibrillary collagen formation, tumor stiffness and cancer metastasis to lymph nodes and lungs (Gilkes et al., 2013b).

Function of PLOD2 in lung cancer progression differs slightly from breast cancer; ectopic expression of PLOD2 enhances both primary cancer growth and metastasis (Chen et al., 2015). PLOD2 hydroxylates telopeptidyl lysine residues on collagen, subsequently increasing the level of hydroxylysine aldehyde–derived collagen cross-links (HLCCs) and lowering levels of lysine aldehyde–derived cross-links in lung cancer tissue (Chen et al., 2015). Recent study also reveal that PLOD2 expression induces PI3K/AKT signaling in glioma (Song et al., 2017) and non-small-cell lung cancer (NSCLC) (Du et al., 2017); activation of the PI3K pathway may contribute to increased cell proliferation, migration and invasion.

It is well established that PLOD2 protein locates in ER (Liefhebber et al., 2010). However, a recent study shows that PLOD2 protein can be secreted by lung cancer cells and induce collagen remodeling (Chen et al., 2016). Addition of recombinant PLOD2 to the extracellular space promotes HLCC formation in the extracellular matrix, suggesting that secreted PLOD2 is functional (Chen et al., 2016). However, function of secreted PLOD2 in cancer development and progression remains to be determined.

Cancer associated fibroblasts (CAFs) and stellate cells, as the major source of ECM production in the tumor microenvironment, promote tumor cell invasion and migration through the PLOD2-induced collagen cross-link (Bozóky et al., 2013; Pankova et al., 2016). PLOD2 is highly expressed in CAFs; silencing PLOD2 expression in CAFs significantly reduced the tumor invasion and metastasis (Pankova et al., 2016). Knockdown of PLOD2 in pancreatic stellate cells inhibits directional migration of cancer cells within the matrices by constructing an insensitive microenvironment of three-dimensional (3D) matrices to tumor migration (Sada et al., 2016). These results indicate that PLOD2 expressed in stromal cells is crucial for cancer progression.

## Future direction

Loss of function mutations and abnormal PLOD expression are involved in many collagen-related diseases. Impairment of PLOD1 function contributes the development of Kyphoscoliotic type of EDS. Mutations of PLOD3 cause the connective tissue disorder (Salo et al., 2008). Many studies demonstrate that increased PLOD2 and PLOD3 expression is required for cancer progression and metastasis. Therefore, targeting PLOD is a potential therapeutic strategy for cancer and other collagen-related diseases. Unfortunately, there is no potent PLOD inhibitor available. Since the client protein and function of PLOD1, PLOD2, and PLOD3 in collagen synthesis are different, it is important to develop specific inhibitors for PLOD to halt cancer progression. Another strategy to inhibit PLOD activity in cancer tissue is to reduce PLOD expression; therefore, further understanding how PLOD is regulated during cancer development may identify signaling pathways to target PLOD.

## Author contributions

All authors listed have made a substantial, direct and intellectual contribution to the work, and approved it for publication.

### Conflict of interest statement

The authors declare that the research was conducted in the absence of any commercial or financial relationships that could be construed as a potential conflict of interest.

## Abbreviations

PLOD: procollagen-lysine 2-oxoglutarate 5-dioxygenase
LH: lysyl hydroxylases
EDS: Ehlers-Danlos syndrome
RDEB: recessive dystrophic epidermolysis bullosa
BMD: bone mineral density
GT: galactosyltransferase
GGT: galactosylhydroxylysine -glucosyltransferase
BS: Bruck Syndrome
bAVM: brain arteriovenous malformations
HIF-1α: hypoxia-inducible factor-1α
EMT: Epithelial-Mesenchymal Transition
HIF-1: hypoxia-inducible factor 1
ACHP: 2-Amino-6-[2-(cyclopropylmethoxy)-6-hydroxyphenyl]-4-(4-piperidinyl)−3 pyridinecarbonitrile
BMP-2: bone morphogenetic protein-2
AT-MSCs: adipose tissue-derived mesenchymal stem cells
ESCC: esophageal squamous-cell carcinoma
HCC: hepatocellular carcinoma
HLCCs: hydroxylysine aldehyde–derived collagen cross-links
NSCLC: non-small-cell lung cancer
ER: endoplasmic reticulum
CAFs: Cancer associated fibroblasts
ECM: extracellular matrix
3D: three-dimensional.
